# Dermatological manifestations of hematologic neoplasms. Part I: secondary specific skin lesions^☆^

**DOI:** 10.1016/j.abd.2022.06.002

**Published:** 2022-11-04

**Authors:** Patricia Karla de Souza, Rafael Oliveira Amorim, Letícia Siqueira Sousa, Mariana Dias Batista

**Affiliations:** a Hospital Israelita Albert Einstein, São Paulo, SP, Brazil; b Department of Dermatology, Universidade Federal de São Paulo, São Paulo, SP, Brazil

**Keywords:** Leukemia, myeloid, Lymphoma, Plasmacytoma, Skin neoplasms

## Abstract

Cutaneous manifestations occur during the course of hematologic malignancies and precede, follow, or are late events in relation to the diagnosis. They result from paraneoplastic phenomena, tumor infiltrations, and immunosuppression resulting from the hematologic neoplasia itself or its treatment. The dermatologist must be aware of these conditions, which can help both in the diagnosis of the underlying disease and in the reduction of patient morbidity. This review (part I) addresses skin lesions associated with direct infiltration by systemic hematologic malignancies.

## Introduction

Hematologic malignancies constitute a group of neoplasms with extremely heterogeneous clinical and behavioral characteristics that lead to aggressive or indolent, acute or chronic conditions, with different prognoses and involvement of different organs.^1^ The skin may be involved in a specific way, through infiltration by malignant cells, or non-specific, as in paraneoplastic dermatoses, in alterations common to hematological disorders, such as pallor and ecchymosis, among others, and as those related to treatment and opportunistic infections.^1^, ^2^ Cutaneous involvement substantially impacts the quality of life of the hematologic patient, in addition to compromising the prognosis in different cases.^1^

Specific skin lesions secondary to systemic hematologic malignancy infiltration (Part 1) and the most common paraneoplastic skin diseases associated with hematologic systemic neoplasms (Part 2) will be discussed. Primary cutaneous lymphomas will not be addressed. Table 1 briefly describes the reviewed conditions and their treatment and Table 2 the epidemiological data of these conditions, according to the literature.

**Table 1:** Specific cutaneous manifestations of hematologic neoplasms: associations and management.

Dermatological diagnosis	Most frequently found hematologic alteration	Typical dermatological manifestation	Management
Leukemia cutis	AML	Firm, smooth, brownish, erythematous, and/or violaceous nodules, plaques, and papules	Anti-leukemia pharmacological treatment
	Others: CLL, AMML, MDS, ALL, T-cell lymphoma/leukemia		
		Erosions, ulcerations, occasional desquamation	Allogeneic BMT
		Trunk, extremities, face	Rescue with DLI (donor lymphocyte infusion) in post-allogeneic BMT recurrence
		Mucosa: gingival hyperplasia	
			Radiotherapy
Cutaneous plasmacytoma	MM	Erythematous-violaceous, isolated, smooth, rounded nodules	Treatment of the underlying disease +/- Radiotherapy
			Cutaneous primary: Radiotherapy; Surgery followed by radiation or chemotherapy
Secondary cutaneous lymphomas	T/NK line	Nodules (60% of cases)	Treatment of underlying lymphoma
	Other: Mature B-line, immature hematologic malignancies, Hodgkin's lymphoma	Macules, patches and plaques	
		Ulceration (9% of cases)	
		T/NK line: multiple/widespread lesions; poor prognosis

**Table 2:** Epidemiological data of the specific cutaneous manifestations of hematologic neoplasms.

Dermatological Diagnosis	Epidemiological data
Leukemia cutis (prevalence)	AML– 10%‒15%
	CLL – 4%‒27%
	ALL – 1%‒3%
	CLM – 2%

Cutaneous plasmacytoma (prevalence)	MM – 4%
Secondary cutaneous lymphomas (relative frequency)	T/NK line – 48%‒72%
	Mature B line – 21%‒25%
	Immature hematologic neoplasms – 4%‒8%
	Hodgkin’s lymphoma – 0%‒4%

## Specific skin lesions secondary to systemic hematologic malignancy infiltration

### Leukemia cutis

Leukemias are neoplastic proliferations of leukocytes and their precursors in the bone marrow and peripheral blood.^3^, ^4^ They are myeloid or lymphoid in origin and, according to cell maturation, acute or chronic.^5^, ^6^ Leukemia cutis (LC) is the extramedullary cutaneous manifestation caused by the infiltration of leukemic cells into the epidermis, dermis, or subcutaneous tissue.^3^, ^7^ It appears before, during, or after the manifestation of the systemic disease as an extramedullary occurrence of the initial disease, as the first manifestation of the hematologic disease, or, rarely, as the primary disease. 7, 8 The rare cases that occur before bone marrow or peripheral blood involvement, whose systemic involvement may take months to years to appear, are called aleukemic leukemias.^9^, ^10^

Leukemia cutis most frequently affects individuals with acute myeloid leukemia (AML); however, it is also seen in chronic myeloid leukemia (CML), acute lymphocytic leukemia (ALL), chronic lymphocytic leukemia (CLL), myelodysplastic syndrome (MDS), T-cell lymphoma/leukemia (TLL), and rarely, in hairy cell leukemia (HCL) and plasma cell leukemia (PCL).^3^, ^4^, ^6^, ^8^^,^^11^

The frequency reported in the literature varies from 2.1% to 30%, depending on the type of primary leukemia, but accurate and robust data are lacking.^3^, ^4^ In different studies, there is also a problem in defining the terminology regarding the terms leukemia cutis, myeloid sarcoma, granulocytic sarcoma, and chloroma, which interferes with the epidemiological data. Myeloid/granulocytic sarcoma is described in the central nervous system, gastrointestinal tract, lymph nodes, testes, ovaries, bones and peritoneum, in addition to the skin. Its occurrence is linked to leukemic diseases of the myeloid cell line, mainly AML, but it also occurs in CML, MDS, and other myeloproliferative disorders.^6^, ^12^ The word “chloroma”, historically described in the 19^th^ century, comes from the Greek word *chloros*. which means green, due to the greenish aspect of the tumor caused by myeloperoxidase oxidation.^13^ The terms granulocytic/myeloid sarcoma emerged around 1965, but they do not contemplate the actual origin of the tumor, which occurs in hematopoietic tissue;^3^ therefore, the WHO in 2016, defined myeloid sarcoma as a tumor mass of myeloid blasts with or without maturation, which occurs at any anatomical site other than the bone marrow. The term chloroma remains in use although not all cases have granules containing myeloperoxidase.^13^ This review follows the recommendations given by Vega et al., where leukemia cutis is a broad term that encompasses the cutaneous infiltrations of any type of leukemia, including myeloid sarcoma, granulocytic sarcoma, or chloroma.^14^

Considering the different types of leukemias, a prevalence of LC of 10% to 15% is observed in AML,^2^, ^3^, ^8^, ^9^ of which the myelomonocytic (AMML) and monocytic (MoAL) subtypes are the most affected, in up to half of the cases.^2^, ^5^, ^6^ In CLL, the most common presentation of systemic leukemias, LC is reported in 4% to 27% of these cases,^3^, ^15^, ^16^ more frequently in the Richter’s syndrome, which is the rare transformation of CLL into a large cell lymphoma.^15^ Regarding the other types of leukemia, data show the involvement of 1% to 3% in ALL and 2% in CML.^9^

The pathogenetic mechanism of skin invasion by leukemic cells is not well understood, suggesting that chemokine receptors and adhesion molecules play a crucial role. The role of cytogenetic alterations related to this type of tumor is also being studied.^2^, ^6^, ^11^

**Figure 1:**
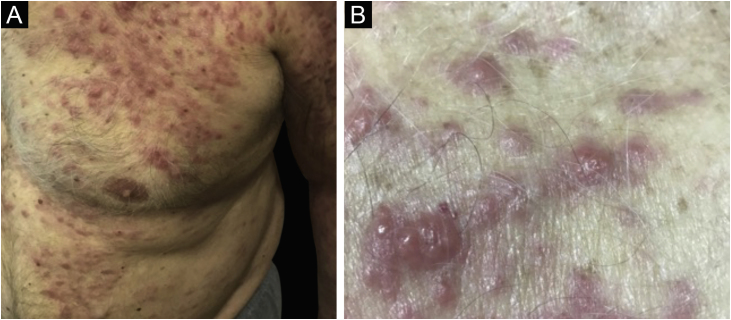
Leukemia cutis in a patient with AML. (A), Multiple erythematous, infiltrated papules and nodules with a smooth surface affecting the trunk and upper limb. (B) Lesions at higher magnification.

**Figure 2:**
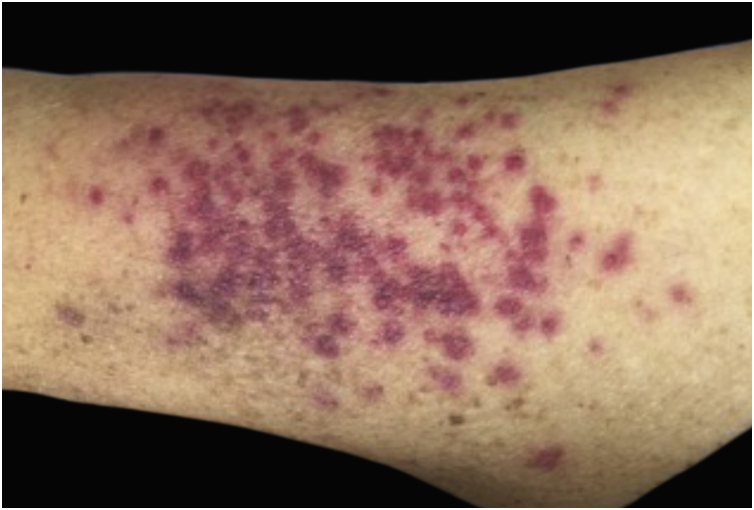
Leukemia cutis in a patient with AML ‒ confluent purpuric papules and some isolated ones with a rough surface on the lateral aspect of the left arm.

**Figure 3:**
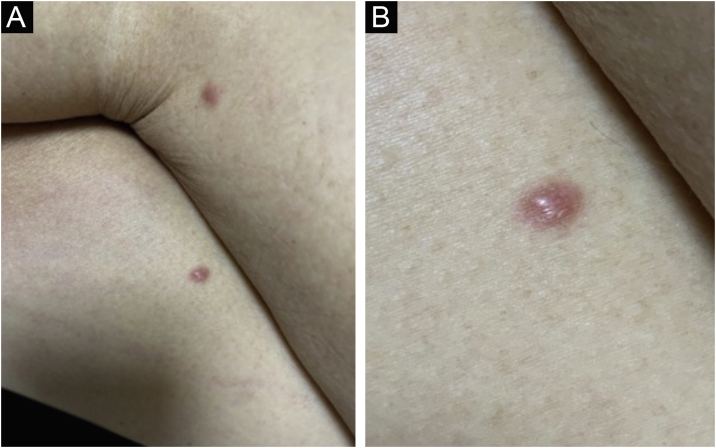
Leukemia cutis in a patient with AML. (A) Post-allogeneic BMT recurrence – two isolated erythematous, smooth, hardened papules on both thighs. (B), Detail of the lesion at higher magnification.

Clinically, the lesions are not pathognomonic, as they are polymorphic, single or, mainly, multiple (Fig. 1).^7^, ^10^ The disease does not have a preferred location, despite being more commonly described on the trunk, extremities, and face,^6^, ^8^ but it is rarely seen in the palmoplantar region.^3^ Some authors mention that the infiltration occurs preferentially in sites with previous skin inflammation or infection.^8^, ^14^, ^16^ Lesion morphology does not allows the diagnosis of the involved cell line.^2^, ^3^, ^6^ They present as nodules, plaques and papules of varied consistency, are usually firm, of various sizes, and are brownish, erythematous, and/or violaceous, often purpuric in color (Fig. 2) due to the accompanying thrombocytopenia.^2^, ^4^ The surface is usually smooth, (Fig. 3), but erosions, ulcerations, and the presence of desquamation are sometimes observed.^3^ Other clinical presentations of leukemia cutis are very rare, such as maculopapular exanthema, exfoliative erythroderma, and single, rarely multiple, ulcers.^3^, ^4^, ^7^ Atypical presentations have been described in isolated cases, such as psoriasiform lesions, leonine facies, figured macules, papulovesicular lesions.^3^, ^17^ The aleukemic form is described as diffuse and papulonodular.^6^ More than 80% of the skin lesions are asymptomatic, few patients report pain or pruritus.^4^ The location and distribution also do not correlate with any specific cell type of leukemia cutis;^10^, ^12^ however, some authors associate the generalized conditions with acute forms of leukemia, while solitary, clustered, or dispersed lesions may be seen in chronic or acute forms.^3^ The dynamics of the appearance of infiltrations are also related to the type of leukemia, with the ones with rapid onset, in outbreaks, being associated with acute forms, while the ones with gradual onset are associated with chronic forms. In CLL, specific cutaneous lesions appear in the later stages of the disease, approximately 40 months after the first systemic manifestations.^15^, ^16^

The oral mucosa can be affected and gingival hyperplasia is the most frequently observed clinical condition, often hemorrhagic with evolution to necrosis.^3^, ^4^ This type of mucosal involvement is seen especially in AML and AMML. Oral ulcers, papules, and nodules are rarely seen in CLL and other leukemias.^3^

Leukemia cutis is seen in 1/3 of systemic leukemia cases in childhood, mainly in the congenital forms. 4, 18, 19 AML is the type of leukemia most often associated with LC in this age group.^18^ In congenital leukemia, the lesions have a “blueberry muffin” appearance in 30% of cases.^14^

When correlating the temporality of the cutaneous involvement onset in relation to the systemic one, in 55% to 77% of the cases LC lesions appear in cases already diagnosed with leukemia,^4^ and only in 23% to 38% of cases they appear concomitantly with systemic manifestations.^3^, ^7^, ^8^ In a recent study, Yook et al. (2022) reported that 71% to 100% of LC cases appear at or after the diagnosis of systemic leukemia.^9^

Skin biopsy is the reference examination for diagnosis, which is based on histopathological evaluation, considering the pattern of distribution, cytological findings, and immunohistochemical characteristics.^3^, ^10^, ^12^ Cytological characteristic vary with the type of underlying leukemia.^10^ The infiltrate is perivascular and/or peri-adnexal, nodular or diffuse, occupying mainly the deep dermis and subcutaneous tissue, with necrotic cells, mitotic figures, and nuclear pleomorphism.^3^, ^14^, ^17^ Immunohistochemical analysis helps to identify the cell line, especially in cases of diagnostic doubt regarding cutaneous lymphoma. Myeloid alterations are diagnosed by the absence of specific T and B-cell markers and by the expression of myelomonocytic markers such as CD68, CD43, CD33, lysozyme, myeloperoxidase, CD117, and CD15.^16^ LC in CLL is characterized by the co-expression of CD19, CD5, CD20, CD79 and CD23.^16^ Immunohistochemical findings should always be correlated with bone marrow and peripheral blood findings, in addition to molecular genetic studies.^6^, ^10^ In cases of no history of leukemia, the diagnosis can be difficult, as the cells may be poorly differentiated and diagnostic difficulty occurs, confusing it with non-Hodgkin's lymphoma.^6^, ^12^ Imaging studies collaborate in cases of subcutaneous nodules, contributing to establishing lesion location, assessing the number of lesions, and differential diagnoses.^20^

There is no consensus on the treatment of leukemia cutis. Systemic treatment is aimed at treating the underlying disease and there are few randomized trials that specifically assess the response. The choice of protocol depends on the cell line involved, immunohistochemical characteristics, and time of onset in relation to systemic disease and cytogenetic alterations.^10^ The time of evolution is crucial in this choice and one should also consider whether or not the case is a recurrence. Based on this knowledge, traditional anti-leukemia chemotherapy, consolidation with allogeneic bone marrow transplantation (BMT), or rescue with donor lymphocyte infusion (DLI) in post-allogeneic BMT are chosen.^4^, ^12^ Radiotherapy that addresses the lesion locally can also be used, especially in an isolated lesion, in recurrence after BMT, or in cases where symptoms caused by the tumor, due to compression, need to be quickly relieved.^4^, ^6^

Several studies associate the presence of leukemia cutis with an unfavorable prognosis when compared to the overall survival rate for systemic disease.^2^, ^7^, ^12^ Considering the chronic disease, the onset of LC demonstrates the presence of the blast phase, which suggests progression to the acute form.^14^, ^21^ Possible cytogenetic abnormalities detected through karyotype and fluorescence *in situ* hybridization (FISH) studies demonstrate aggressive behavior.^17^ Existing data indicate that survival after one year is very low, based on studies with small sample sizes.^6^, ^17^ In the study by Chang et al., 74.3% of LC cases died within one year and the median survival was 7.2 months.^4^ Yook et al. (2022) demonstrated that 84% of the patients died after the LC diagnosis, of which 93% of them died within 10 months.^9^ LC-associated survival in AML and AMML cases is as low as four months.^3^ A 2019 cohort study shows that patients with AML and leukemia cutis are 2.06 times more likely to die than patients without skin infiltration.^21^ Data on CLL show that the prognosis is associated with the histological characteristics, being poor ‒ 49% survival at 2 years ‒ when there are more than 5% of large B lymphocytes in the cutaneous infiltrate and favorable – 97% survival at 2 years ‒ when there are more than 95% small B lymphocytes.^16^ Recent studies demonstrate that in the absence of systemic disease progression, such as for Richter's syndrome, leukemia cutis in CLL does not lead to worsening of the prognosis.^15^ However, the literature is still scarce and conflicting when assessing leukemia cutis and the prognosis of the underlying disease.^6^

### Cutaneous plasmacytoma

Plasma cell dyscrasias are characterized by the clonal neoplastic expansion of cells that secrete monoclonal immunoglobulins. The clinical spectrum is diverse and groups entities of different degrees of severity, from asymptomatic monoclonal gammopathy of uncertain significance (MGUS) to malignancies of greater clinical severity, such as multiple myeloma (MM).^22^

Cutaneous plasmacytoma is a rare neoplasm of plasma cells that infiltrates the skin, either by direct involvement, by contiguity of a close focus, or at a distance, via a hematogenous or lymphatic route.^22^, ^23^ The most often described cutaneous plasmacytomas arise in the context of MM, usually as a late complication.^24^ There are few reports in the literature, occurring in less than 4% of cases, although some authors believe that underdiagnosis occurs since in autopsy data of patients with MM the number of extramedullary cutaneous involvement is much higher.^24^, ^25^, ^26^ There is a much rarer form, primary cutaneous plasmacytoma, which occurs without evidence of another medullary or extramedullary plasma cell disease, currently classified in the group of marginal zone lymphomas. Cutaneous plasmacytomas are associated with all immunoglobulin classes, with the exception of IgE, and those related to IgG are the most frequently observed.^23^

**Figure 4:**
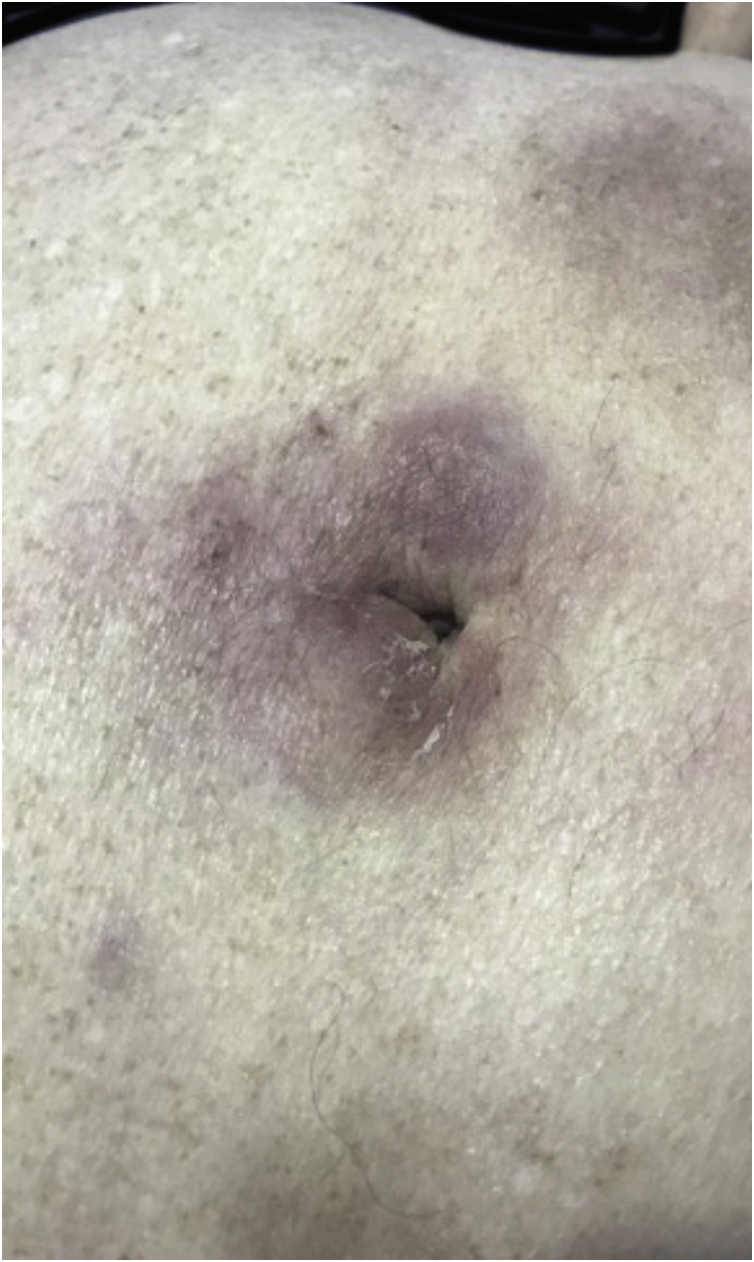
Cutaneous plasmacytoma. Erythematous-violaceous nodules, poorly defined and infiltrated in the periumbilical region.

The most representative clinical form is isolated, smooth, rounded erythematous-violaceous nodules (Fig. 4), but there have been descriptions of flesh-colored nodules in rare cases.^26^ There is no difference in the clinical picture according to the immunoglobulin involved.

Studies demonstrate that the tumor microenvironment is the main regulator of the metastatic process in the extramedullary involvement of plasma cell neoplasms, but the exact mechanism is not yet understood.^26^

A biopsy with histopathological and immunohistochemical analyses confirms the diagnosis. A nodular plasma cell infiltrate is the main identified pattern, followed by the interstitial one. The presence of immunoreactivity for CD138 is constant, in addition to the frequent absence of expression of CD45 and CD20.^24^, ^26^

Prognosis is poor and the mean survival time is 8.5 months.^24^, ^25^ Deletion of the RB1 gene has been reported to be associated with worse prognosis.^24^

Treatment of plasmacytomas with systemic involvement is that of the underlying disease, with or without associated radiotherapy. Primary cutaneous plasmacytomas are treated with radiotherapy alone, or by surgery followed by localized radiotherapy and/or chemotherapy.^25^

### Secondary cutaneous lymphomas

Cutaneous lymphomas can be subdivided into two broad groups: primary, in which there is no evidence of extracutaneous disease at the time of diagnosis, and those that are secondary to systemic lymphoma.^27^ Primary cutaneous lymphomas (PCL) represent a heterogeneous group of entities and will not be addressed in this review.

Secondary cutaneous lymphomas (SCL) account for 20% to 50% of cutaneous lymphomas.^28^ They can be classified, according to the cell line that originated them, as T/NK lymphoma or B lymphoma.^28^ Among the reported cases of SCL in the literature, the relative frequency of the mature T/NK cell line ranged from 48% to 72% in different studies, whereas the mature B line ranged from 21% to 45%, immature hematological neoplasms corresponded to 4% to 8% of cases and Hodgkin's lymphoma accounted for 0% to 4% of cases.^28^, ^29^, ^30^, ^31^, ^32^

**Figure 5:**
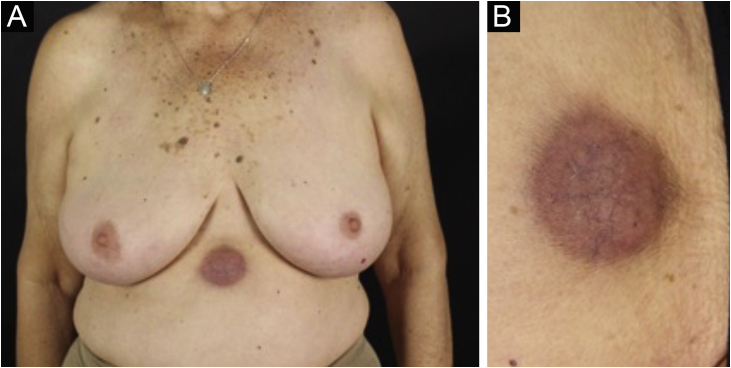
Secondary cutaneous follicular lymphoma. (A) Brownish-erythematous, infiltrated subcutaneous tumor, with telangiectasias in the epigastric region. (B) Lesion at higher magnification.

The clinical manifestations of SCL are polymorphic. Nodules are often present (Fig. 5) in up to 60% of cases, but macules, patches, and plaques can also occur. Ulceration occurs in up to 9% of cases. Lesion morphology did not correlate with survival in one case series.^28^

Patients with T/NK line SCL more often manifest multiple or disseminated lesions and the presence of these lesions is a poor prognostic factor.^28^ Peripheral T-cell lymphomas may present clinically as nodules or tumors, but generalized maculopapular or urticarial eruptions have been described, with lesion morphology varying in the same patient over time.^33^

**Figure 6:**
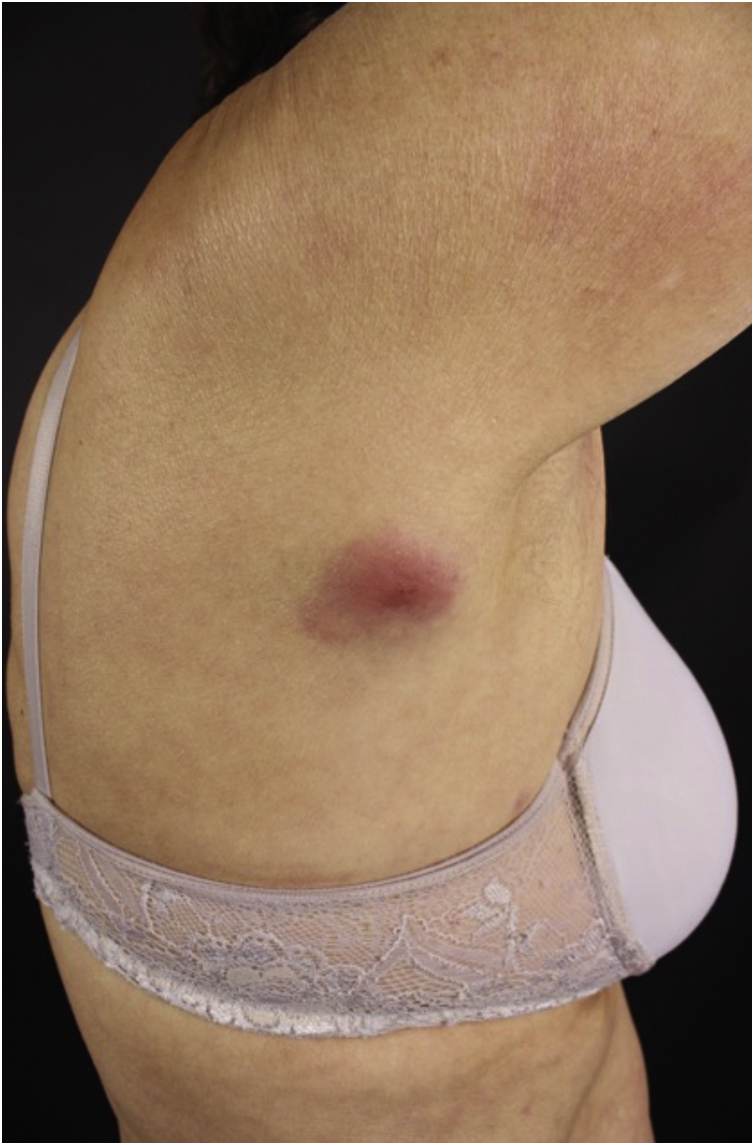
Secondary cutaneous CD30+ anaplastic lymphoma – erythematous, infiltrated, raised, reddish plaque in the central portion on the posterior right axillary line.

Regarding CD30+ anaplastic lymphomas, there are also significant differences between the primary cutaneous form and the secondary form. The secondary CD30+ anaplastic cutaneous lymphoma (Fig. 6) is more often characterized by papules and nodules, has a higher frequency of B symptoms, and has a worse prognosis, with higher mortality and shorter five-year survival.^34^

Hodgkin's lymphoma is associated with specific cutaneous manifestations in less than 1% of cases, usually in advanced refractory disease.^35^ The mode of spread is by direct extension of neoplastic cells to the skin.^36^ Skin involvement by direct extension has also been described in high-grade nodal B lymphomas.^37^

The diagnosis of SCL depends on the diagnosis of the associated nodal lymphoma. The diagnosis of lymphomas is a complex one and includes clinical, morphological, histopathological, immunohistochemical, and molecular factors.^38^ The immunophenotype of CD20 and CD79a expression identifies B-cell line lymphomas, while the CD3 marker, as well as CD2, CD7, and LAT markers, identify the T-cell line. Other markers used for the diagnosis and classification of lymphomas are the panlymphocytic marker CD45, the NK cell marker CD56, and other cytotoxic markers for T and NK cells. Very often, only clinical, histopathological, and immunohistochemical factors are not enough to establish the diagnosis of lymphoma. In these cases, molecular methods are also used to investigate clonality and identify the cell line involved.^38^ To differentiate between PCL and SCL, it is necessary to perform the patient’s staging through imaging tests, bone marrow biopsy, peripheral blood flow cytometry, or other methods, aiming to assess the presence of nodal or other organ involvement at the time of diagnosis, which characterizes the SCL.

When skin involvement occurs within the first six months of diagnosis, the prognosis is worse than in cases in which these lesions occur six months after the diagnosis.^28^ When compared to PCL, SCL have worse five-year survival. The five-year survival for SCL is 31%, while for PCL it ranges from 87% to 92.5%.^39^

Treatment of SCL comprises the treatment of the nodal lymphoma that caused it. Radiotherapy alone is chosen for the localized forms or combined with chemotherapy for aggressive disease. For B-lymphomas, the anti-CD20 antibody rituximab is frequently used, together or not with multidrug chemotherapy. Other treatments used in refractory lymphomas include brentuximab vedotin, ibrutinib, acalabrutinib and idelalisib. Immunotherapy with checkpoint inhibitors has also been associated in recent years in the therapeutic arsenal used to treat lymphomas.^40^ Bone marrow transplantation is an alternative for refractory cases. CAR-T (chimeric antigen receptor T) cells have also shown promising results in refractory hematologic malignancies.^41^

## Conclusion

In part I of this review, the authors demonstrate to dermatologists, hematologists, and clinicians the importance of a complete dermatological examination and familiarity with the skin alterations, which may represent a neoplastic infiltration of an already known condition or even a lesion that precedes the diagnosis of systemic disease. Etiological elucidation always needs to be confirmed by a skin biopsy with anatomopathological and immunohistochemical examination. The identification of this type of extramedullary lesion helps in the decision-making related to treatment, which, if performed early, may change prognosis.

More epidemiological work is required in this area of ​​knowledge, aiming to obtain robust and reliable statistical data. It is important to highlights the need for multidisciplinary team working for this complex type of patient, in which the dermatologist is the specialist responsible for the evaluation and diagnosis of skin alterations.

## Financial support

None declared.

## Authors' contributions

Patricia Karla de Souza: Design of the study; drafting of the manuscript and critical review of important intellectual content; design of the study together with the co-authors; critical review of the literature; approval of the final version of the manuscript.

Rafael Oliveira Amorim: Drafting of the manuscript and critical review of important intellectual content; design of the study together with the co-authors; critical review of the literature; approval of the final version of the manuscript.

Letícia Siqueira Sousa: Drafting of the manuscript and critical review of important intellectual content; design of the study together with the co-authors; critical review of the literature; approval of the final version of the manuscript.

Mariana Dias Batista: Drafting of the manuscript and critical review of important intellectual content; design of the study together with the co-authors; critical review of the literature; approval of the final version of the manuscript.

## Conflicts of interest

None declared.
